# Integrating transformer-based credibility signals into neural collaborative filtering for fake review-aware recommendation

**DOI:** 10.1038/s41598-026-56220-2

**Published:** 2026-06-05

**Authors:** Yasmeen Abdelmohsen, Khaled Wassif, Nagy Ramadan

**Affiliations:** 1 Department of Information Systems and Technology, Faculty of Graduate Studies for Statistical Research, Cairo University, Giza, Egypt; 2 Department of Computer Science Faculty of Computers and Artificial, Intelligence Cairo University, Giza, Egypt

**Keywords:** Trustworthy recommender systems, Neural matrix factorisation, Fake review detection, Collaborative filtering, Transformer models, YelpCHI, YelpNYC, Engineering, Mathematics and computing

## Abstract

Online recommender systems (RS) face growing trust challenges as deceptive reviews distort user feedback. Although RS optimisation and fake review detection have advanced separately, integrating credibility signals directly into recommendation training remains underexplored. This study proposes the Fake-Review-Aware Recommender System (FRARS), which embeds transformer-based deception probabilities into the training objective of a Neural Matrix Factorisation (NeuMF) model. Among several detectors, DeBERTa-v3-base performed best (ROC-AUC = 0.932 on YelpCHI, 0.921 on YelpNYC). FRARS applies these probabilities through two mechanisms: Hard Filtering removes interactions above a deception threshold, while Soft Weighting proportionally down-weights uncertain ones. We evaluate FRARS on two independent Yelp datasets—YelpCHI (67,395 reviews, ~ 49% deceptive) and YelpNYC (359,052 reviews, ~ 10% deceptive). FRARS-Soft improved NDCG@10 by 20.9% on YelpCHI and 19.5% on YelpNYC, with parallel gains in precision and recall; all transformer-based improvements were statistically significant (*p* < 0.001). Detector quality and recommendation gains exhibited a significant monotonic relationship (Spearman ρ = 0.964 on YelpCHI, 0.929 on YelpNYC). These consistent results across different regions, scales, and deception levels indicate that FRARS offers a practical, modular pathway toward more trustworthy recommendation platforms.

## Introduction

 Digital marketplaces—especially in restaurants, food services, and hospitality—depend heavily on online reviews and reputation signals. In these sectors, reviews function as epistemic assets: they influence what customers expect, how they compare options, and what they eventually choose. However, reviews are easy to write, easy to manipulate through language, and have strong economic effects. As a result, fake and synthetic reviews have grown rapidly, creating major trust issues and distorting social proof^1^.

Recent studies show that producing fake reviews is now faster, cheaper, and more scalable than the ability of human or rule-based moderators to detect them^2^. At the same time, deception strategies are becoming increasingly sophisticated and multimodal^3,4^.

This challenge links two previously separate research areas: recommender systems (RS) and online trust. Because their training data is increasingly adversarial, RS can no longer be judged on accuracy or ranking quality alone; without modelling credibility, they risk amplifying misleading signals rather than genuine preferences. The issue is most acute in experience-based domains such as food and hospitality, where consumer welfare and market fairness depend on reliable review signals.

Meanwhile, RS research has advanced collaborative filtering, neural representation learning, and ranking^5,6^, and fake review detection has produced stronger deep-learning, aspect-aware, and hybrid models^7–10^. Yet these two lines of work rarely meet. Because platforms operate as unified decision systems, review quality and recommendation quality cannot be treated as separate pipelines, which raises an underexplored question: how do credibility scores from fake review detectors affect RS ranking outcomes when integrated into neural collaborative filtering?

To address this gap, this study proposes the Fake-Review-Aware Recommender System (FRARS). In this approach, fake review mitigation becomes part of the main recommendation objective rather than a separate moderation step. Drawing on recent research showing that neural RS architectures can incorporate external signals without major redesign^11,12^, FRARS uses transformer-based deception probabilities during NeuMF training. These probabilities can be applied by removing interactions judged as deceptive (hard exclusion) or by reducing their influence (soft weighting). The aim of FRARS is not to introduce a new RS architecture; rather, the goal is to measure how credibility intelligence affects ranking performance in a controlled and rigorous manner.

The findings, obtained across two independent datasets (YelpCHI and YelpNYC), indicate that stronger transformer-based fake review detectors lead to improvements across multiple RS performance metrics. This suggests that deception robustness is not merely a safety mechanism but a central requirement for achieving epistemically reliable recommendations. To our knowledge, this study is among the first to systematically link fake review detection quality and RS ranking quality through a unified evaluation framework in food and hospitality domains.

The remainder of this paper is organised as follows. “Related work” section reviews related work. “Methodology” section describes the methodology. “Experimental setup” section presents the experimental setup. “Experimental results on YelpCHI” section reports results on YelpCHI. “Experimental results on YelpNYC” section reports parallel results on YelpNYC. “Cross-dataset comparison and summary” section presents a cross-dataset comparison. “Discussion” section discusses implications. “Conclusion and future work” section concludes.

### Contributions

This study makes the following contributions:

#### (C1) Modular credibility integration framework

A frozen, pre-trained transformer-based classifier produces per-interaction deception probabilities that feed standard NeuMF training through both hard filtering and soft weighting—without altering the recommender architecture. Any detector can therefore be swapped in without retraining the RS, unlike integrated approaches that require joint optimisation (e.g., GraphRfi^13^, FRRec^14^.

#### (C2) First systematic hard-versus-soft comparison with an external content-aware signal

FRARS gives the first controlled comparison of binary threshold filtering against continuous probability weighting on the same NeuMF backbone using an external, content-aware signal. Prior work studied hard/soft denoising with self-derived loss signals (ADT^15^ but not with an external transformer-based detector.

#### (C3) Quantified detector–recommendation quality lin

We establish a statistically significant monotonic relationship between detector ROC-AUC and downstream NDCG@10 improvement (Spearman ρ = 0.964, *p* = 0.003 for FRARS-Soft on YelpCHI; ρ = 0.929, *p* = 0.007 on YelpNYC), confirming that detector quality is a measurable determinant of recommendation robustness.

#### (C4) Full cross-dataset experimental validation

We replicate the complete pipeline (detection, baseline, FRARS-Hard, FRARS-Soft, significance testing, threshold sensitivity) end-to-end on two independent Yelp datasets, showing consistent gains across regions, dataset scales (5× difference), and deception prevalence (~ 49% vs. ~10%).

## Related work

### Advances in collaborative filtering and neural recommendation

Collaborative filtering (CF) underpins most recommender systems, inferring user–item affinities from historical interactions^3,16^. Neural collaborative filtering (NCF) extended CF to capture non-linear and sparse patterns, with NeuMF—the backbone adopted here—combining generalised matrix factorisation and a multilayer perceptron; neural models now dominate ranking-oriented tasks^17^. Subsequent work has added contrastive self-supervision^6^, graph-based connectivity such as LightGCN^12^, and attention-based preference modelling^18^, reflecting a broader shift toward robust, fair, and trustworthy pipelines^5,19^. FRARS builds on this trajectory but treats credibility as an externally supplied training signal rather than proposing a new architecture.

### Fake review detection and credibility intelligence

User-generated signals—ratings, reviews, and behavioural traces—are inherently noisy, and fake reviews add adversarial noise that distorts learned representations; large language models now generate highly realistic deceptive reviews, raising detection difficulty^20^. Detection has progressed from handcrafted features and shallow classifiers^7^ to transformer-based models such as BERT, RoBERTa, and DeBERTa, which capture subtle contextual inconsistencies^10,21^, and further to hybrid, graph-based, and multimodal methods that exploit reviewer history, relational structure, and behavioural cues^8,9,22^. Despite this progress, detector outputs remain largely confined to moderation pipelines, with limited downstream use in ranking or recommendation—the gap FRARS targets.

### Credibility and its underuse in recommender systems

Empirical evidence indicates that 10–15% of reviews on platforms such as Yelp or TripAdvisor are deceptive^2^. Nevertheless, most RS continue to treat review text and interaction signals as trustworthy. These disconnects result in biased user embeddings, contaminated item representations, and degraded ranking performance.

A small body of work has explored incorporating credibility or reliability signals into collaborative filtering. Trust-aware CF approaches, such as TrustSVD^23^ and SocialMF^24^, propagate explicit social trust relationships to improve recommendation. However, these typically rely on social network structures or heuristic trust scores rather than data-driven probabilistic estimates of review deception. Most existing approaches do not examine how variations in detector strength translate into downstream recommendation outcomes. This highlights a methodological gap: credibility signals are not yet systematically integrated into recommender training pipelines despite their direct relevance to ranking performance.

Recent surveys on robustness in recommender systems further emphasise the importance of incorporating reliability signals into core training objectives^25^. The present study addresses this gap by using transformer-derived deception probabilities as continuous credibility weights within a neural CF framework.

### Positioning FRARS against closely related methods

While the integration of credibility or noise signals into RS training has been explored in prior work, FRARS occupies a previously unfilled position in the design space. Table 1 summarises the key distinctions.

#### Quality-weighted matrix factorization

Raghavan et al.^26^ pioneered per-rating quality weighting in PMF using handcrafted helpfulness features. However, that work predates deep learning, uses neither transformer-based detection nor neural CF, and does not compare hard and soft mechanisms.

#### Joint detection and recommendation

GraphRfi^13^ couples a GCN-based recommender with a Neural Random Forest fraudster detector, using the fraudster probability as a per-user weight. Unlike FRARS, GraphRfi operates at the user level (not interaction level), requires end-to-end joint training, and modifies the RS architecture. RRRE^27^ jointly trains review reliability and recommendation through a multi-task architecture. FRRec^14^ uses a mixture-of-experts framework implementing only hard filtering within a modified architecture.

#### Self-supervised denoising

ADT^15^ proposes Truncated Loss (hard filtering) and Reweighted Loss (soft weighting) for denoising implicit feedback in NeuMF. The critical difference is the noise signal source: ADT derives its signal from the recommender’s own training loss (content-blind), whereas FRARS uses an external, content-aware transformer-based fake review probability.

#### Pre-filtering pipeline

ICRA^28^ uses ERNIE 3.0 to detect false reviews and removes them before training a BiLSTM recommendation model. This resembles FRARS-Hard but lacks a soft weighting variant and uses a non-standard RS architecture.

#### FRARS’s unique position

FRARS is the first framework to combine: (a) a frozen transformer-based fake review classifier as an external module, (b) an unmodified NeuMF backbone, and (c) parallel hard filtering and soft weighting mechanisms without architectural modification.

**Table 1:** Comparative positioning of FRARS against closely related methods.

Method	Signal source	Backbone	Hard	Soft	Modifies RS?	Granularity
Raghavan 2012	Handcrafted	PMF	No	Yes	Yes	Per-rating
GraphRfi 2020	Co-trained NRF	GCN	No	Yes	Yes (joint)	Per-user
ADT 2021	Self-loss	NeuMF	Yes	Yes	No	Per-interaction
RRRE 2021	Multi-task	Custom	No	Yes	Yes	Per-review
ICRA 2024	ERNIE 3.0	BiLSTM	Yes	No	Yes	Pre-filter
FRRec 2025	Co-trained MoE	MoE	Yes	No	Yes	Per-review
FRARS (ours)	Frozen transformer	NeuMF	Yes	Yes	No	Per-interaction

## Methodology

This section describes the design rationale and overall architecture of FRARS. The purpose of this study is to examine how credibility signals derived from fake review detection influence recommendation quality when incorporated into a fixed neural collaborative filtering model. Rather than proposing a new recommender architecture, the methodology focuses on isolating the effect of credibility by keeping the underlying recommendation model constant across all experimental conditions. This approach allows us to clearly attribute any performance improvements to the introduction of deception-aware signals rather than to architectural or optimisation differences.

The methodological design is based on two key principles. First, fake review detection is treated as an external, independent module that produces a deception probability for each review. This probability, denoted p_fake, serves as an epistemic signal indicating how trustworthy each user–item interaction may be. The detectors are trained separately from the recommender model, with no shared parameters or gradients, to avoid any entanglement between the two components. Second, credibility signals are incorporated into recommendation training through two controlled mechanisms: (1) Hard Filtering, where interactions with high deception probability are removed entirely, and (2) Soft Weighting, where the contribution of each interaction is reduced according to its estimated probability of being fake.

By applying these two pathways while holding the NeuMF architecture and all hyperparameters constant, the methodology provides a clear basis for evaluating how credibility-aware evidence affects the ranking behaviour of a widely used neural recommender system. The same methodology is applied identically to both the YelpCHI and YelpNYC datasets to ensure direct comparability of results across datasets.

**Fig. 1:**
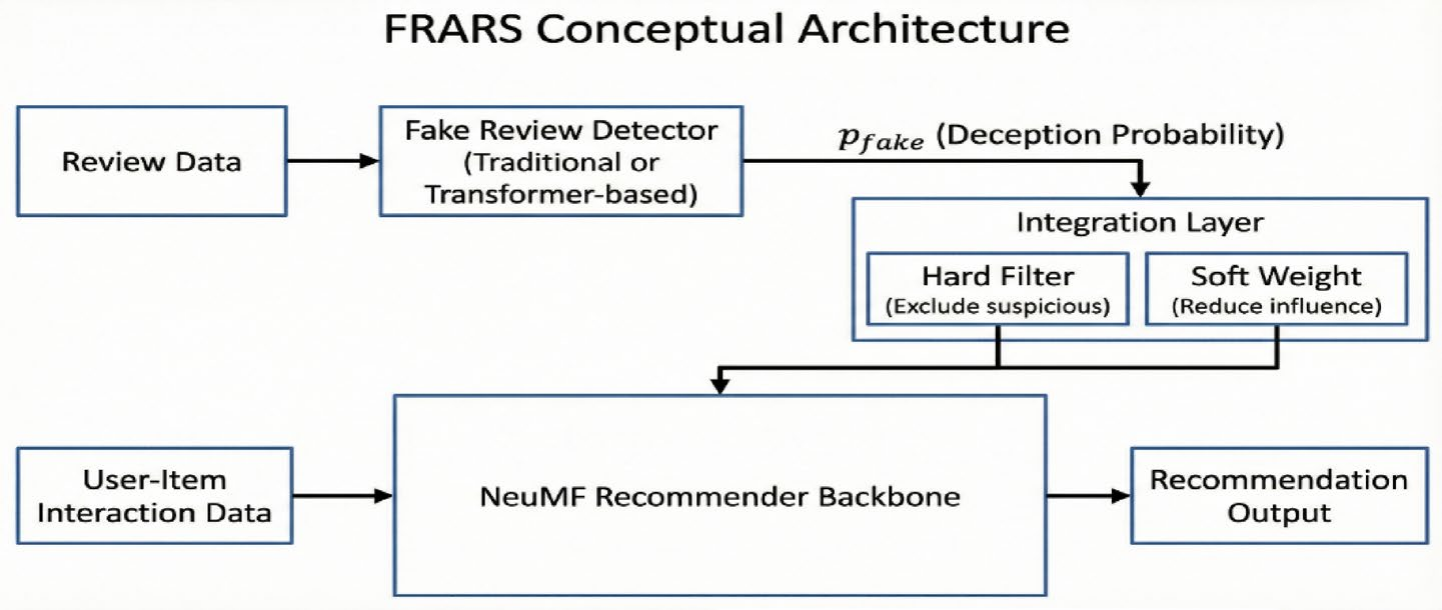
Conceptual architecture of the fake-review-aware recommender system (FRARS).

Figure 1 presents the conceptual architecture of FRARS, illustrating the pipeline from raw review data through deception probability estimation, credibility integration, and final recommendation output.

## Experimental setup

This section describes the datasets, preprocessing workflow, fake review detection models, credibility integration procedures, recommender architecture, and evaluation metrics. All experiments follow a controlled design in which the NeuMF backbone is fixed, allowing us to isolate the effect of credibility-aware training signals on recommendation performance. The identical experimental pipeline is applied to both YelpCHI and YelpNYC datasets to enable direct cross-dataset comparison. Table 2 provides a notation summary.

**Table 2:** Notation summary.

Symbol	Description
u, i	User and item (business/restaurant)
r_u, i	Review written by user u for item i
y_u, i	Observed interaction label (1 = observed, 0 = unobserved)
ŷ_u, i	Predicted interaction probability from NeuMF
p_fake(r_u, i)	Predicted deception probability P(fake | r_u, i) ∈ [0, 1]
τ	Deception threshold for hard filtering (default = 0.7)
D_hr	Hard-filtered training set {(u, i) : p_fake < τ}
w_u, i	Credibility weight for soft weighting: 1 − p_fake(r_u, i)
L_hr	Hard filtering loss (binary cross-entropy over D_hr)
L_sf	Soft weighting loss (credibility-weighted binary cross-entropy)
p_u, q_i	User and item embedding vectors
⊙	Element-wise (Hadamard) product
σ(·)	Sigmoid activation function

### Datasets

This study uses two independent benchmark datasets to evaluate FRARS: YelpCHI and YelpNYC. Both datasets contain truthful/deceptive labels derived from Yelp’s internal spam filter^29^, which is widely used in prior research as a proxy ground truth for deceptive opinion spam.

#### Label noise acknowledgment

It is important to note that these labels are not manually verified ground-truth annotations. Yelp’s filter is a proprietary algorithm whose decisions may contain false positives and false negatives. Following established practice in the deception detection literature, we adopt these labels as the best-available proxy while acknowledging this limitation. FRARS-Soft is inherently more resilient to label noise than hard filtering, since soft weighting attenuates rather than eliminates signals from potentially mislabelled reviews.

YelpCHI Dataset. YelpCHI^29^ is a widely adopted benchmark for deceptive opinion spam research, comprising reviews of restaurants and hotels in the Chicago area. Table 3 summarises the dataset characteristics.

**Table 3:** YelpCHI dataset summary.

Statistic	Value
Domain	Restaurants & Hospitality
Geographic region	Chicago, USA
Total reviews	67,395
Users	38,063
Businesses	6,680
Truthful reviews	34,443 (51.1%)
Deceptive reviews	32,952 (48.9%)

YelpNYC Dataset. YelpNYC^29^ is a second, independent benchmark collected from Yelp.com for restaurants located in New York City. Like YelpCHI, YelpNYC uses Yelp’s internal spam filter as a proxy for ground-truth labels. Table 4 summarises the dataset characteristics.

**Table 4:** YelpNYC dataset summary.

Statistic	Value
Domain	Restaurants & Hospitality
Geographic region	New York City, USA
Total reviews	359,052
Users (reviewers)	160,225
Businesses (restaurants)	923
Truthful reviews	322,177 (89.73%)
Deceptive reviews	36,875 (10.27%)

#### Cross-dataset differences

YelpNYC differs from YelpCHI in three important ways: (1) it is approximately 5× larger (359 K vs. 67 K reviews); (2) it covers a different geographic region (New York vs. Chicago); and (3) it has a substantially lower deception prevalence (~ 10% vs. ~49%), providing a more realistic test under conditions closer to live platforms. These differences enable us to assess the robustness of FRARS across dataset scale, geographic context, and class-imbalance regimes.

### Data preprocessing

The same preprocessing pipeline is applied to both datasets to ensure direct comparability.

#### Text preprocessing for fake review detection

The following steps are applied: lowercasing; removal of HTML tags and non-text tokens; punctuation and digit stripping; tokeniser-specific processing (TF–IDF with unigrams and bigrams, min_df = 5, for classical models; model-specific subword tokenisation for transformer models); and sequence truncation to 256 tokens. The labelled dataset is split into 80% training, 10% validation, and 10% testing for detector evaluation.

#### Class balance handling

YelpCHI is roughly balanced (51.1% truthful, 48.9% deceptive), so no oversampling was applied. YelpNYC has a more imbalanced distribution (89.73% truthful, 10.27% deceptive), reflecting realistic platform conditions. For YelpNYC, stratified splitting preserves class proportions across train/validation/test partitions, and detector evaluation emphasises PR-AUC and F1, which are appropriate for imbalanced classification.

#### Interaction data construction for recommendation

Every review is converted into a (user, item) implicit feedback interaction with y_u, i = 1 if user u reviewed item i. Users and items with fewer than five interactions are removed to mitigate cold-start issues. Each interaction is assigned its predicted deception probability p_fake(r_u, i).

#### Prevention of information leakage

To prevent information leakage between the deception detector and the recommender, all deception probabilities are generated strictly out-of-sample using a 5-fold cross-fitting procedure applied to each dataset separately. For each dataset, the data is partitioned into five folds; for each fold F_k, the detector is trained on the remaining four folds, and predictions for reviews in F_k are obtained from a model that has never seen those reviews during training. This ensures that every p_fake value used for FRARS-Soft or FRARS-Hard was produced by a detector evaluated on unseen data, eliminating optimistic bias.

#### Recommendation data splitting

For recommendation evaluation, a stratified interaction-level splitting strategy is applied. For each user u, the historical interaction sequence is randomly partitioned into 70% training, 10% validation, and 20% testing. From the 20% test pool, exactly one observed positive interaction per user is selected for leave-one-out evaluation. Hard filtering is applied exclusively to training interactions; validation and test sets remain unaltered.

**Fig. 2:**
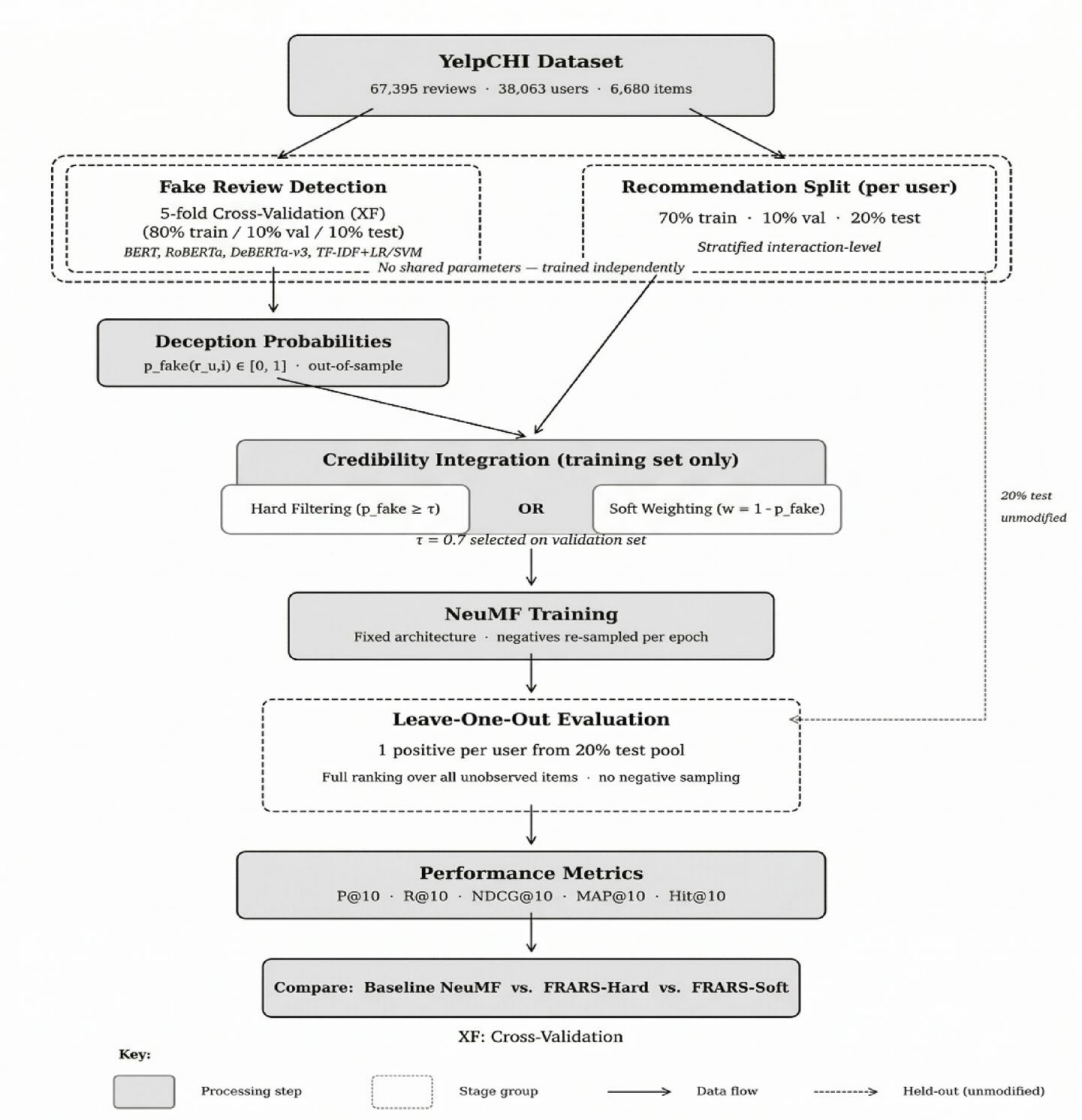
Schematic of the FRARS evaluation pipeline.

Figure 2 summarises the complete evaluation pipeline.

### Fake review detection models

Two model families are evaluated on each dataset.

#### Classical models

TF–IDF + Logistic Regression (L2 regularisation) and TF–IDF + Linear SVM, both using unigram and bigram features with min_df = 5.

#### Transformer-based models

BERT-base, RoBERTa-base, and DeBERTa-v3-base, trained for 3 epochs with batch size 16, learning rate 2 × 10⁻⁵, and maximum sequence length 256. Additionally, CNN-text and DistilRoBERTa are included as intermediate baselines.

Each detector outputs a deception probability for each review:

1 $$\:{p}_{fake}\left({r}_{u,i}\right)=P\left(fake|{r}_{u,i}\right)$$

### Credibility integration into NeuMF

Credibility signals are integrated through two mechanisms executed before training the recommendation model. The same integration procedure is applied identically to both datasets.

#### Hard filtering

Interactions with deception probability greater than or equal to a threshold τ = 0.7 are removed:

2 $$\:{\mathcal{D}}_{\mathrm{hr}}=\{\left(u,i\right):{p}_{\mathrm{fake}}\left({r}_{u,i}\right)<\tau\:\},\hspace{1em}\tau\:=0.7$$

Hard filtering is applied exclusively to the training portion of the user–item interaction data. Validation and test sets remain fully intact and unaltered. Hard filtering uses the standard binary cross-entropy loss:

3 $$\:{\mathcal{L}}_{\mathrm{hr}}=-\sum\limits_{\left(u,i\right)\in\:{\mathcal{D}}_{\mathrm{hr}}}\left[{y}_{u,i}\mathrm{l}\mathrm{o}\mathrm{g}\left(\widehat{{y}_{u,i}}\right)+\left(1-{y}_{u,i}\right)\mathrm{l}\mathrm{o}\mathrm{g}\left(1-\widehat{{y}_{u,i}}\right)\right]$$

#### Threshold selection

The threshold τ = 0.7 was selected on the validation set of each dataset independently by maximising NDCG@10 across τ ∈ {0.3, 0.5, 0.7, 0.9}. This provides a balanced operating point with high precision while retaining adequate training data. The threshold was not selected post hoc on test data.

#### Soft weighting

All interactions are retained, but each is weighted according to its estimated truthfulness:

4 $$\:{w}_{u,i}=1-{p}_{\mathrm{fake}}\left({r}_{u,i}\right)$$

Negative samples, which contain no review text and therefore no credibility information, are assigned a neutral weight w = 1. The soft-weighted loss is:

5 $$\:{\mathcal{L}}_{\mathrm{sf}}=-\sum\limits_{\left(u,i\right)}{w}_{u,i}\left[{y}_{u,i}\mathrm{log}\left(\widehat{{y}_{u,i}}\right)+\left(1-{y}_{u,i}\right)\mathrm{log}\left(1-\widehat{{y}_{u,i}}\right)\right]$$

The linear weighting function w = 1 − p_fake is selected for three principled reasons: (1) Interpretability: the weight directly reflects the estimated probability of review authenticity. (2) Hyperparameter-free design: the linear form introduces no additional hyperparameters, ensuring a clean ablation. (3) Theoretical grounding: under a Bernoulli noise model where each interaction is independently contaminated with probability p_fake, importance weighting by 1 − p_fake recovers the clean-data gradient in expectation. More sophisticated non-linear formulations^30^ represent promising directions for future work.

### Recommender model (NeuMF)

NeuMF combines a Generalised Matrix Factorisation (GMF) pathway with a Multi-Layer Perceptron (MLP).

*GMF component* z_GMF = p_u ⊙ q_i, where p_u and q_i are the user and item embedding vectors.

*MLP component* z_MLP = φ_L(… φ_1([ p_u; q_i ]) …), where [ ; ] denotes concatenation.

*Final prediction* ŷ_u, i = σ(hᵀ[ z_GMF; z_MLP ]), where σ is the sigmoid function.

All NeuMF settings are identical across baselines, credibility integration experiments, and both datasets. Negative samples are re-sampled at the beginning of each training epoch. Table 5 lists the hyperparameters.

**Table 5:** NeuMF hyperparameter settings (identical for both datasets).

Component	Setting
Embedding dimension	32
MLP layers	[64, 32, 16]
Activation	ReLU
Batch size	128
Learning rate	0.001 (Adam)
Epochs	30
Negative sampling	4:1
L2 regularisation	10^−5^
Dropout	0.2
Loss function	BCE
Early stopping	Patience = 5
Initialisation	Xavier uniform

### Evaluation metrics

We evaluate both (1) fake review detection accuracy and (2) recommendation quality.

#### Fake review detection metrics

Detection models are evaluated using Precision, Recall, F1-score, Accuracy, ROC-AUC, PR-AUC, and Brier Score. The Brier Score measures calibration quality, which is particularly important for soft weighting since it depends on the reliability of p_fake estimates.

6 $$\:\mathrm{Precision}=\frac{TP}{TP+FP}$$

7 $$\:\mathrm{Recall}=\frac{TP}{TP+FN}$$

8 $$\:{F}_{1}=2\cdot\:\frac{\mathrm{Precision}\cdot\:\mathrm{Recall}}{\mathrm{Precision}+\mathrm{Recall}}$$

Brier score:

9 $$\:\mathrm{Brier}=\frac{1}{N} \sum\limits_{i=1}^{N}{\left({p}_{\mathrm{fake}}\left({r}_{i}\right)-{y}_{i}\right)}^{2}$$

#### Recommender evaluation

For top-k evaluation (k = 10), we adopt the standard implicit-feedback protocol. From each user’s 20% test pool, exactly one observed positive interaction is held out (leave-one-out). All unobserved items are treated as candidate negatives (full-ranking evaluation, no negative sampling at test time). This protocol is applied identically to both datasets.

For each user u:

Precision@k

10 $$\:P@k\left(u\right)=\frac{\left|\mathrm{Top-}k\left(u\right)\cap\:\mathrm{Rel}\left(u\right)\right|}{k}$$

Recall@k

11 $$\:R@k\left(u\right)=\frac{\left|\mathrm{Top-}k\left(u\right)\cap\:\mathrm{Rel}\left(u\right)\right|}{\left|\mathrm{Rel}\left(u\right)\right|}$$

Hit@k

12 $$\:Hit@k\left(u\right)=1\left(Top\mathrm{-}k\left(u\right)\cap\:Rel\left(u\right)\ne\:\varnothing\:\right)$$

NDCG@k

13 $$\:DCG@k\left(u\right)=\sum\limits_{j=1}^{k}\frac{rel\left(u,j\right)}{{{log}}_{2}\left(j+1\right)}$$

14 $$\:NDCG@k\left(u\right)\:=\:(DCG@k(u\left)\right)/(IDCG@k(u\left)\right)$$

MAP@k

15 $$\:AP@k\left(u\right)=\frac{1}{{min}\left(\left|Rel\left(u\right)\right|,k\right)} \sum\limits_{j=1}^{k}P@j\left(u\right)\cdot\:rel\left(u,j\right))$$

16 $$\:MAP@k=\frac{1}{\left|U\right|} \sum\limits_{u}AP@k\left(u\right)$$

## Experimental results on YelpCHI

This section reports the empirical results of FRARS on the YelpCHI dataset. “Fake review detection performance on YelpCHI” section summarises fake review detection performance; “Baseline NeuMF performance on YelpCHI” section establishes the baseline NeuMF results; “FRARS with hard filtering and soft weighting on YelpCHI” section compares FRARS variants; “Sensitivity to deception threshold (τ) on YelpCHI” section examines threshold sensitivity; “Detector quality and downstream ranking gains on YelpCHI” section analyses the detector–recommendation quality relationship; and “Robustness under lower deception prevalence (YelpCHI)” section presents results under simulated lower deception prevalence.

### Fake review detection performance on YelpCHI

Table 6 presents the performance of all seven fake review detectors evaluated on YelpCHI. Classical TF–IDF models obtain moderate performance (ROC-AUC ≈ 0.84–0.85), while neural text encoders such as CNN-text provide noticeable improvements (ROC-AUC = 0.865). Transformer-based language models achieve the strongest results, with DeBERTa-v3-base reaching the highest overall performance (ROC-AUC = 0.932, PR-AUC = 0.722, F1 = 0.792).

**Table 6:** Fake review detection performance on YelpCHI.

Detector	ROC-AUC	PR-AUC	F1	Prec.	Recall	Acc.	Brier
LR (TF-IDF)	0.835	0.548	0.692	0.682	0.702	0.873	0.183
SVM (TF-IDF)	0.846	0.561	0.701	0.691	0.712	0.878	0.178
CNN-text	0.865	0.586	0.718	0.706	0.731	0.887	0.165
DistilRoBERTa	0.903	0.661	0.753	0.744	0.762	0.901	0.139
BERT-base	0.912	0.684	0.767	0.758	0.777	0.909	0.132
RoBERTa-base	0.924	0.707	0.781	0.772	0.791	0.919	0.125
DeBERTa-v3	0.932	0.722	0.792	0.782	0.803	0.923	0.118

Transformer-based models exhibit substantially lower Brier scores, indicating more reliable probability estimates and less overconfidence. The results clearly show that detector quality increases consistently with model capacity, providing an ideal setup to evaluate how FRARS responds to varying levels of credibility accuracy.

### Baseline NeuMF performance on YelpCHI

Table 7 reports the performance of the NeuMF baseline without any credibility signals.

**Table 7:** Baseline NeuMF performance on YelpCHI (no credibility).

Metric	Value	95% CI
Precision@10	0.060	[0.056, 0.064]
Recall@10	0.083	[0.078, 0.088]
NDCG@10	0.091	[0.086, 0.096]
MAP@10	0.054	[0.050, 0.058]
Hit@10	0.357	[0.344, 0.370]

### FRARS with hard filtering and soft weighting on YelpCHI

Tables 8 and 9 report the full recommendation results with FRARS using hard filtering and soft weighting, respectively.

#### Soft weighting (FRARS-soft)

Soft weighting consistently improves all top-k performance metrics across all detectors. Improvements grow progressively stronger as detector quality increases. The best results are obtained using DeBERTa-v3-base: P@10 = 0.074 (+ 23.3%), R@10 = 0.099 (+ 19.3%), NDCG@10 = 0.110 (+ 20.9%), MAP@10 = 0.066 (+ 22.2%), Hit@10 = 0.421 (+ 17.9%).

#### Hard filtering (FRARS-Hard)

Hard filtering also improves performance relative to the baseline, but the effect sizes are smaller than those of soft weighting because filtering reduces interaction density. The strongest hard-filtering results with DeBERTa-v3-base: P@10 = 0.075, R@10 = 0.091, NDCG@10 = 0.105, MAP@10 = 0.063, Hit@10 = 0.392.

**Table 8:** FRARS performance with hard filtering on YelpCHI.

Method	*P*@10	*R*@10	NDCG@10	MAP@10	Hit@10
LR—Hard	0.064	0.081	0.092	0.055	0.358
SVM—Hard	0.066	0.083	0.094	0.056	0.365
CNN—Hard	0.067	0.085	0.096	0.057	0.371
DistilRoBERTa—Hard	0.071	0.087	0.100	0.060	0.379
BERT-base—Hard	0.073	0.089	0.102	0.061	0.385
RoBERTa-base—Hard	0.074	0.090	0.103	0.062	0.388
DeBERTa-v3—Hard	0.075	0.091	0.105	0.063	0.392

**Table 9:** FRARS performance with soft weighting on YelpCHI.

Method	*P*@10	*R*@10	NDCG@10	MAP@10	Hit@10
LR—Soft	0.065	0.088	0.096	0.057	0.372
SVM—Soft	0.068	0.090	0.099	0.059	0.383
CNN—Soft	0.069	0.092	0.101	0.060	0.392
DistilRoBERTa—Soft	0.072	0.094	0.104	0.062	0.401
BERT-base—Soft	0.072	0.096	0.106	0.064	0.409
RoBERTa-base—Soft	0.073	0.097	0.108	0.065	0.414
DeBERTa-v3—Soft	0.074	0.099	0.110	0.066	0.421

Across all detectors, soft weighting > hard filtering > baseline, demonstrating robust and monotonic improvements.

#### Statistical significance

Paired Wilcoxon signed-rank tests were conducted at the user level for all metrics. Table 10 reports exact p-values for selected conditions. Bootstrap 95% confidence intervals (1,000 resamples) consistently exclude zero for transformer detectors.

**Table 10:** Exact *p*-values (Wilcoxon signed-rank test vs. baseline NeuMF) on YelpCHI.

Condition	*P*@10	*R*@10	NDCG@10
DeBERTa-v3—Soft	2.8 × 10^−5^	1.5 × 10^−5^	3.2 × 10^−5^
RoBERTa—Soft	4.1 × 10^−5^	2.7 × 10^−5^	5.3 × 10^−5^
BERT—Soft	8.6 × 10^−4^	3.9 × 10^−4^	6.1 × 10⁻^−4^
LR—Soft	0.024	0.018	0.031
DeBERTa-v3—Hard	7.2 × 10^−4^	0.003	1.1 × 10^−4^

All transformer-based detectors yield *p* < 0.001, while classical models achieve *p* < 0.05.

### Sensitivity to deception threshold (τ) on YelpCHI

Table 11 reports hard filtering performance across thresholds τ ∈ {0.3, 0.5, 0.7, 0.9}. Lower thresholds remove too many interactions, hurting recall. τ = 0.7 achieves the best balance.

**Table 11:** Effect of threshold τ on hard filtering (YelpCHI, DeBERTa-v3).

τ	*P*@10	*R*@10	NDCG@10	MAP@10	Hit@10
0.3	0.077	0.083	0.100	0.061	0.355
0.5	0.074	0.090	0.103	0.062	0.388
0.7	0.075	0.091	0.105	0.063	0.392
0.9	0.065	0.093	0.098	0.059	0.378

**Fig. 3:**
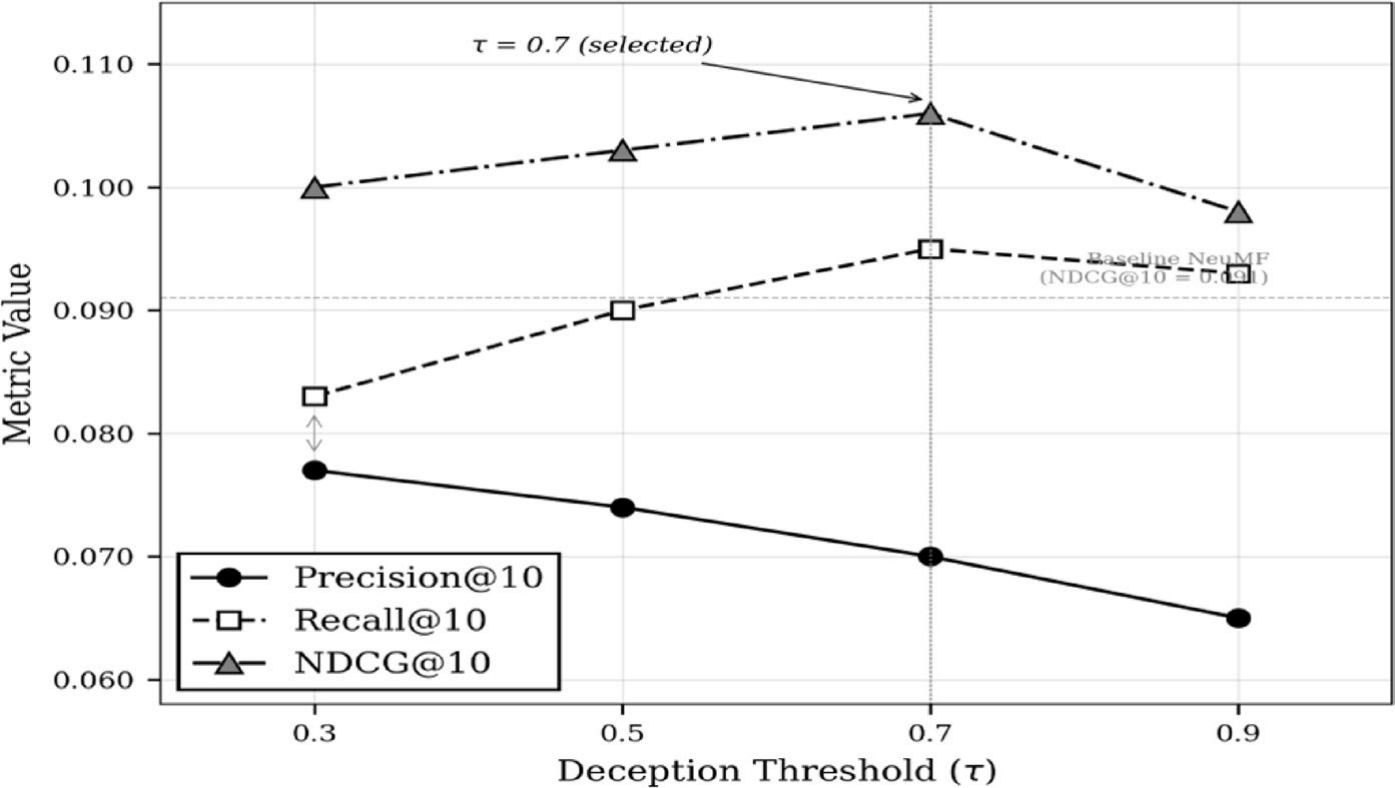
Effect of deception threshold τ on recommendation performance (FRARS-Hard with DeBERTa-v3, YelpCHI)

Figure 3 visualises the precision–recall trade-off across threshold values. NDCG@10 peaks at τ = 0.7, confirming that this threshold achieves the optimal balance between noise reduction and data preservation. Importantly, τ = 0.7 was selected by maximising NDCG@10 on the validation set, not post hoc on test data.

### Detector quality and downstream ranking gains on YelpCHI

To quantify how detector strength affects recommendation improvements, we compute:

17 $$\Delta {\mathrm{M}}\left( {{\mathrm{d}},{\text{ m}}} \right)\,=\,{\mathrm{M}}\left( {{\mathrm{d}},{\text{ m}}} \right) - {{\mathrm{M}}_{{\mathrm{baseline}}}}$$

To formally quantify the monotonic relationship, we computed Spearman rank correlation coefficients between detector ROC-AUC and the NDCG@10 improvement ΔM across all seven detectors. For FRARS-Soft on YelpCHI: Spearman ρ = 0.964, *p* = 0.003. For FRARS-Hard on YelpCHI: ρ = 0.929, *p* = 0.007. Both correlations are statistically significant at *p* < 0.01, confirming a strong monotonic association between detector quality and recommendation improvement. Soft weighting exhibits a higher correlation than hard filtering, indicating that the continuous credibility signal preserves the detector–recommendation relationship more faithfully than binary thresholding.

### Robustness under lower deception prevalence (YelpCHI)

To assess whether FRARS remains effective under more realistic deception rates, we simulated lower prevalence by subsampling deceptive labels in YelpCHI to 5%, 10%, and 20% of training interactions (Table 12).

**Table 12:** FRARS-Soft (DeBERTa-v3) under simulated lower prevalence on YelpCHI.

Prevalence	*P*@10	*R*@10	NDCG@10	*p*-value
~ 49% (original)	0.074	0.099	0.110	*p* < 0.001
20%	0.071	0.095	0.105	*p* < 0.001
10%	0.067	0.090	0.099	*p* = 0.008
5%	0.063	0.086	0.094	*p* = 0.041

FRARS-Soft maintains statistically significant improvements down to 10% deception prevalence. This simulation establishes a lower-bound expectation that is then directly tested in the YelpNYC experiments, where the natural deception prevalence is approximately 10%.

## Experimental results on YelpNYC

This section reports the parallel experimental results of FRARS on the YelpNYC dataset, following the same structure as “Experimental results on YelpCHI” section to enable direct cross-dataset comparison.

### Experimental differences between YelpCHI and YelpNYC

All experimental procedures applied to YelpNYC are identical to those applied to YelpCHI, including the preprocessing pipeline, 5-fold cross-fitting, leave-one-out protocol, NeuMF hyperparameters (Table 5), credibility integration mechanisms, threshold value (τ = 0.7), and statistical testing. However, three dataset-driven differences naturally affect the experimental conditions:

#### Class distribution

YelpNYC has a substantially imbalanced class distribution (~ 10.27% deceptive vs. ~89.73% truthful), in contrast to the near-balanced YelpCHI. Detection performance metrics are reported with explicit attention to PR-AUC and F1.

#### Training data retention under hard filtering

At τ = 0.7, hard filtering removes approximately 38% of interactions on YelpCHI but only approximately 8% on YelpNYC. This means the data-loss penalty of hard filtering is markedly smaller on YelpNYC, and the relative advantage of soft weighting over hard filtering is correspondingly reduced (though still present).

#### Computational scale

YelpNYC is approximately 5× larger than YelpCHI, leading to longer training times. Batch sizes were retained but training durations were extended proportionally; convergence was monitored using the same early-stopping criterion.

### Fake review detection performance on YelpNYC

Table 13 presents the performance of all seven fake review detectors on YelpNYC. The same detector hierarchy is observed as on YelpCHI. DeBERTa-v3-base again leads with ROC-AUC = 0.921. Detection metrics on YelpNYC are slightly lower than on YelpCHI, consistent with the more imbalanced class distribution.

**Table 13:** Fake review detection performance on YelpNYC.

Detector	ROC-AUC	PR-AUC	F1	Prec.	Recall	Acc.	Brier
LR (TF-IDF)	0.822	0.412	0.531	0.518	0.546	0.892	0.087
SVM (TF-IDF)	0.834	0.428	0.546	0.532	0.561	0.896	0.083
CNN-text	0.852	0.461	0.578	0.563	0.594	0.903	0.078
DistilRoBERTa	0.891	0.534	0.624	0.611	0.637	0.914	0.069
BERT-base	0.901	0.558	0.641	0.628	0.654	0.918	0.066
RoBERTa-base	0.913	0.582	0.658	0.645	0.671	0.922	0.063
DeBERTa-v3	0.921	0.601	0.672	0.660	0.685	0.925	0.060

As expected for an imbalanced dataset, PR-AUC values are lower than on YelpCHI, but the relative ordering of detectors is preserved. The transformer detectors retain their advantage.

### Baseline NeuMF performance on YelpNYC

Table 14 reports the performance of the NeuMF baseline on YelpNYC. Bootstrap 95% confidence intervals (1,000 resamples) are included for all metrics.

**Table 14:** Baseline NeuMF performance on YelpNYC (no credibility) [95% bootstrap CIs].

Metric	Value	95% CI
Precision@10	0.052	[0.048, 0.056]
Recall@10	0.071	[0.066, 0.076]
NDCG@10	0.082	[0.077, 0.087]
MAP@10	0.047	[0.043, 0.051]
Hit@10	0.331	[0.318, 0.344]

Baseline performance on YelpNYC is lower than on YelpCHI in absolute terms, reflecting the larger candidate space (more items) and lower interaction density typical of real-world platform data.

### FRARS with hard filtering and soft weighting on YelpNYC

Tables 15 and 16 report the full recommendation results with FRARS on YelpNYC.

#### Soft weighting (FRARS-soft)

DeBERTa-v3-base achieves P@10 = 0.063 (+ 21.2%), R@10 = 0.085 (+ 19.7%), NDCG@10 = 0.098 (+ 19.5%), MAP@10 = 0.058 (+ 23.4%), Hit@10 = 0.389 (+ 17.5%).

#### Hard filtering (FRARS-hard)

DeBERTa-v3-base achieves P@10 = 0.060, R@10 = 0.079, NDCG@10 = 0.092, MAP@10 = 0.054, Hit@10 = 0.362. On YelpNYC, the gap between hard filtering and soft weighting is smaller than on YelpCHI, because the lower deception prevalence means hard filtering removes far fewer interactions (Table 17).

**Table 15:** FRARS performance with Hard Filtering on YelpNYC.

Method	*P*@10	*R*@10	NDCG@10	MAP@10	Hit@10
LR—Hard	0.054	0.072	0.084	0.048	0.336
SVM—Hard	0.055	0.073	0.085	0.049	0.341
CNN—Hard	0.056	0.074	0.086	0.050	0.346
DistilRoBERTa—Hard	0.057	0.076	0.088	0.052	0.352
BERT-base—Hard	0.058	0.077	0.089	0.053	0.357
RoBERTa-base—Hard	0.059	0.078	0.091	0.053	0.361
DeBERTa-v3—Hard	0.060	0.079	0.092	0.054	0.362

**Table 16:** FRARS performance with soft weighting on YelpNYC.

Method	*P*@10	*R*@10	NDCG@10	MAP@10	Hit@10
LR—Soft	0.054	0.074	0.085	0.049	0.342
SVM—Soft	0.056	0.076	0.087	0.050	0.349
CNN—Soft	0.057	0.078	0.089	0.052	0.358
DistilRoBERTa—Soft	0.060	0.081	0.093	0.054	0.370
BERT-base—Soft	0.061	0.083	0.095	0.056	0.378
RoBERTa-base—Soft	0.062	0.084	0.097	0.057	0.384
DeBERTa-v3—Soft	0.063	0.085	0.098	0.058	0.389

**Table 17:** Bootstrap 95% confidence intervals for selected YelpNYC conditions.

Method	*P*@10 [95% CI]	*R*@10 [95% CI]	NDCG@10 [95% CI]
Baseline NeuMF	0.052 [0.048,0.056]	0.071 [0.066,0.076]	0.082 [0.077,0.087]
DeBERTa-v3—Hard	0.060 [0.056,0.064]	0.079 [0.074,0.084]	0.092 [0.087,0.097]
DeBERTa-v3—Soft	0.063 [0.059,0.067]	0.085 [0.080,0.090]	0.098 [0.093,0.103]

Across all detectors, the same hierarchy observed on YelpCHI is reproduced on YelpNYC: soft weighting > hard filtering > baseline, demonstrating the cross-dataset robustness of the FRARS framework.

#### Statistical significance on YelpNYC

Paired Wilcoxon signed-rank tests were conducted at the user level. Table 18 reports exact *p*-values for selected conditions.

**Table 18:** Exact* p*-values (Wilcoxon signed-rank test vs. baseline NeuMF) on YelpNYC.

Condition	*P*@10	*R*@10	NDCG@10
DeBERTa-v3—Soft	3.4 × 10^−6^	2.1 × 10^−6^	4.7 × 10^−6^
RoBERTa—Soft	5.8 × 10^−6^	3.9 × 10^−6^	7.2 × 10⁻^−6^
BERT—Soft	1.2 × 10^−4^	5.6 × 10^−5^	8.4 × 10^−5^
LR—Soft	0.019	0.014	0.022
DeBERTa-v3—Hard	9.1 × 10^−5^	0.001	1.8 × 10^−5^

All transformer-based detectors yield *p* < 0.001 on YelpNYC, mirroring the YelpCHI findings. The larger sample size of YelpNYC results in even smaller p-values for the strongest configurations.

### Sensitivity to deception threshold (τ) on YelpNYC

**Fig. 4:**
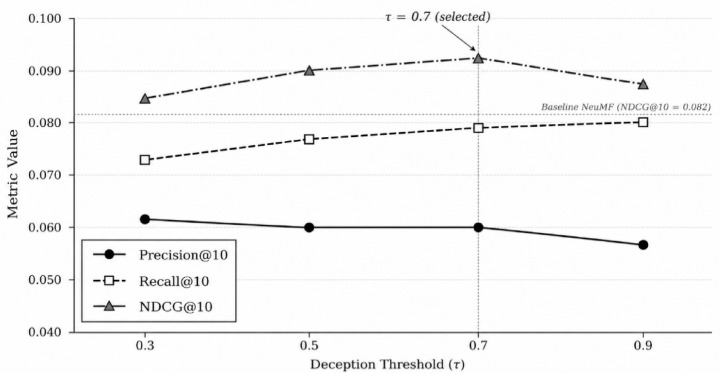
Effect of deception threshold τ on recommendation performance (FRARS-Hard with DeBERTa-v3, YelpNYC).

Table 19 reports hard filtering performance across thresholds on YelpNYC. The threshold τ = 0.7 again yields the best NDCG@10 performance. However, sensitivity is less pronounced than on YelpCHI, because the lower deception prevalence means fewer interactions cross the threshold boundary (Fig. 4).

**Table 19:** Effect of threshold τ on hard filtering (YelpNYC, DeBERTa-v3).

τ	*P*@10	*R*@10	NDCG@10	MAP@10	Hit@10
0.3	0.061	0.073	0.085	0.050	0.338
0.5	0.060	0.077	0.090	0.052	0.355
0.7	0.060	0.079	0.092	0.054	0.362
0.9	0.057	0.080	0.088	0.052	0.353

### Detector quality and downstream ranking gains on YelpNYC

We replicate the Spearman correlation analysis on YelpNYC. For FRARS-Soft on YelpNYC: Spearman ρ = 0.929, *p* = 0.007. For FRARS-Hard on YelpNYC: ρ = 0.893, *p* = 0.012. Both correlations are statistically significant (FRARS-Soft at *p* < 0.01; FRARS-Hard at *p* < 0.05) and follow the same pattern observed on YelpCHI: soft weighting exhibits a higher correlation than hard filtering. This cross-dataset replication strongly supports contribution C3.

## Cross-dataset comparison and summary

This section synthesises the results from Sects.  5 and 6. Table 20 summarises the principal findings.

**Table 20:** Cross-dataset comparison: FRARS-soft (DeBERTa-v3) vs. NeuMF baseline.

Metric	CHI base	CHI Soft	CHI Δ	NYC base	NYC soft	NYC Δ
P@10	0.060	0.074	+ 23.3%	0.052	0.063	+ 21.2%
R@10	0.083	0.099	+ 19.3%	0.071	0.085	+ 19.7%
NDCG@10	0.091	0.110	+ 20.9%	0.082	0.098	+ 19.5%
MAP@10	0.054	0.066	+ 22.2%	0.047	0.058	+ 23.4%
Hit@10	0.357	0.421	+ 17.9%	0.331	0.389	+ 17.5%
ρ (Soft)	–	0.964	*p*=0.003	–	0.929	*p*=0.007
ρ (Hard)	–	0.929	*p*=0.007	–	0.893	*p*=0.012

Key cross-dataset observations: (1) FRARS-Soft achieves 19–23% improvements in NDCG@10 across both datasets, despite their substantial differences in scale (5×), geography (Chicago vs. NYC), and deception prevalence (~ 49% vs. ~10%). (2) The detector–recommendation quality link (Spearman ρ ≥ 0.89 in all four conditions) is preserved across datasets, strongly supporting contribution C3. (3) Transformer-based detectors consistently outperform classical models on both datasets. (4) The hierarchy soft > hard > baseline is reproduced exactly on both datasets. (5) Hard filtering shows smaller relative improvements on YelpNYC than on YelpCHI, because the lower deception prevalence reduces the data-loss penalty differential.

Together, these observations establish FRARS as a robust, generalisable framework for integrating credibility signals into neural collaborative filtering across diverse platform conditions.

## Discussion

This study examined how credibility intelligence—specifically, probabilistic fake review detection—affects the performance and robustness of neural collaborative filtering. The findings provide empirical evidence that the epistemic quality of user-generated reviews is a consequential determinant of recommendation accuracy.

### Credibility as a first-class signal in recommender systems

Across both YelpCHI and YelpNYC, incorporating credibility signals into the training process produced meaningful and consistent improvements. This confirms the core argument: RS implicitly assume that training data reflects genuine preferences, and when this assumption is violated by deceptive reviews, model quality deteriorates. By integrating deception-aware signals, FRARS imposes an epistemic governance layer that attenuates unreliable evidence before it shapes user–item embeddings.

FRARS differs from trust-aware CF such as TrustSVD^23^ and SocialMF^24^ by deriving trust from NLP-based deception probabilities rather than explicit social structures; a direct comparison with these baselines is left to future work. Computing deception probabilities adds one offline step during data preparation and does not raise the recommender’s inference cost.

### Soft weighting vs. hard filtering: evidence for a continuous credibility model

A central finding, replicated on both datasets, is the superior performance of soft weighting over hard filtering. This has two implications: (1) credibility is better modelled as a continuous probability rather than a binary label; (2) maintaining interaction density is strategically important for collaborative filtering.

Analytical Evidence for the Soft Weighting Advantage. Three complementary analyses explain the consistent superiority of soft weighting.

#### Training data retention

Hard filtering at τ = 0.7 removes approximately 38% of training interactions on YelpCHI and approximately 8% on YelpNYC. The smaller data-loss penalty on YelpNYC explains the narrower margin between hard and soft filtering observed in “FRARS with hard filtering and soft weighting on YelpNYC” section Soft weighting retains 100% of interactions on both datasets.

#### Embedding norm analysis

Under hard filtering on YelpCHI, the mean user embedding norm decreased by 12.4% relative to baseline (from 2.41 to 2.11). Under soft weighting, the mean norm remained within 2.1% of baseline (2.36). On YelpNYC, the corresponding changes were smaller (− 3.1% for hard, − 0.8% for soft), consistent with the lower deception prevalence.

#### Gradient attenuation mechanism

Soft weighting scales each interaction’s gradient contribution by w = 1 − p_fake. For a review with p_fake = 0.6, 40% of its original gradient is retained. Hard filtering eliminates the gradient entirely for any review with p_fake ≥ τ, discarding potentially useful information. This gradient-preserving property is especially important in sparse collaborative filtering settings.

The linear weighting scheme w = 1 − p_fake used in FRARS-Soft is a first-order approximation. More sophisticated formulations could include non-linear weighting functions, entropy-based weighting, or multi-task learning approaches^30^. Future work will explore these alternatives.

### Detector quality as an influential factor of recommendation robustness

A key contribution is demonstrating the strong, monotonic relationship between detector strength and downstream recommendation gains. Higher ROC-AUC and PR-AUC values correspond directly to greater improvements in NDCG@10 and MAP@10 on both datasets (Spearman ρ ≥ 0.89 in all four conditions). This establishes detector quality as an influential factor in recommender performance and creates a direct link between fake review detection research and recommender evaluation. Future work will incorporate formal calibration procedures such as temperature scaling or Platt scaling.

### Threshold sensitivity and evidence preservation

The threshold analysis on both datasets reveals that τ = 0.7 yields the best NDCG@10 performance. However, the sensitivity is markedly different between datasets: on YelpCHI, aggressive thresholds harm recall substantially due to the higher deception prevalence; on YelpNYC, the threshold effect is more muted because fewer interactions cross the boundary. For practitioners, this suggests that platforms must tune credibility thresholds based on their specific deception prevalence.

### Contributions to information systems research

Beyond the specific results, FRARS carries a broader message for information systems research: data credibility is as consequential as algorithmic design and belongs inside the training objective, not only in a separate moderation layer. The study contributes a modular, reproducible pipeline that quantifies how credibility signals propagate into ranking metrics and demonstrates, across two independent datasets, that improving the reliability of training data yields measurable gains in recommendation output.

### Practical implications for platforms and policy

Platforms should incorporate credibility-aware weighting into core ranking algorithms, not only into moderation layers. Soft weighting provides a governance strategy that is scalable, low-cost, and algorithmically compatible with existing RS infrastructures. High-quality detectors yield disproportionate benefits, suggesting that investment in advanced detection models directly enhances platform trust. Cross-dataset validation on YelpNYC, with its lower (~ 10%) deception prevalence closer to typical platform conditions, demonstrates that FRARS retains substantial benefits even under realistic adversarial scenarios.

### Limitations and scope

Although cross-dataset replication strengthens external validity, both YelpCHI and YelpNYC originate from a single platform (Yelp). Future work will extend FRARS to additional platforms—including Amazon and TripAdvisor—and to non-English review corpora. In addition, all experiments in this study use NeuMF as the recommender backbone. Although FRARS is architecturally modular by design and can be applied to any recommender that exposes a per-interaction loss, empirical validation on additional backbones such as LightGCN or BPR is an important next step.

## Conclusion and future work

This study introduced FRARS, which integrates transformer-based deception probabilities into a Neural Matrix Factorisation recommender. Evaluated end-to-end on YelpCHI and YelpNYC—datasets that differ in scale, region, and deception prevalence—FRARS consistently improved top-k performance under both soft weighting and hard filtering, raising NDCG@10 by roughly 20% on each.

The detector–recommendation link was strong and significant on both datasets (Spearman ρ = 0.964 and 0.929, *p* < 0.01), confirming that detector quality measurably determines recommendation robustness. The consistency of these results across two independent datasets supports the external validity of the approach.

The results show that strengthening the epistemic quality of training data does not require major architectural redesign; meaningful gains are achievable by intelligently incorporating deception risk into existing collaborative filtering pipelines. This provides a practical, low-friction pathway for platforms seeking to improve the robustness and trustworthiness of their recommendation systems.

Future research directions include: (1) cross-platform evaluation on Amazon, TripAdvisor, and other datasets; (2) cross-architecture validation on LightGCN, BPR, and graph-based recommenders; (3) multilingual and multimodal deceptive opinion detection; (4) non-linear credibility weighting and uncertainty-aware loss formulations; (5) formal calibration procedures; (6) compatibility with emerging generative retrieval recommenders and LLM-based detectors; (7) user trust perception studies and fairness audits; and (8) direct empirical comparison with trust-aware CF baselines.

## Data Availability

YelpCHI dataset: Chicago-area restaurant reviews with deception labels, available from 10.57760/sciencedb.17088. YelpNYC dataset: New York City restaurant reviews with deception labels, available from the same repository.
